# Task Failure in Endurance Sports: A Review

**DOI:** 10.1007/s40279-025-02377-1

**Published:** 2026-04-01

**Authors:** Juan-José Pérez-Díaz, José-Antonio Salas-Montoro, James Hopker, Mikel Zabala

**Affiliations:** 1 Department of Physical Education and Sport, Faculty of Sport Sciences, University of Granada, Cam. de Alfacar, 21, 18071 Granada, Spain; 2 School of Sport & Exercise Sciences, University of Kent, Canterbury, Kent UK; 3 Sport and Health University Research Institute (iMUDS), University of Granada, 18007 Granada, Spain

## Abstract

Task failure in endurance sports represents a complex and multifaceted phenomenon likely arising from the dynamic and intricate interaction between peripheral and central mechanisms. Traditional models of fatigue have long emphasized a gradual and inevitable decline in performance because of metabolic, neuromuscular, and cardiovascular limitations. Research has also highlighted that task failure may not only be a product of this progressive deterioration but could also result from acute and unpredictable disruptions at the central level (e.g., psychological factors such as perceived effort) that compromise the athlete’s ability to sustain effort. This review examines the existing literature on task failure, critically describing what is currently known about the influence of peripheral (physiological processes at the skeletal muscle and neuromuscular junction) and central mechanisms (neural processes in the brain and spinal cord, which may integrate physiological and psychological components). Finally, we identify gaps in the current literature and propose directions for future research to refine our understanding of the interplay between peripheral and central contributors to task failure, emphasizing the need for integrative approaches to optimize endurance performance.

## Key Points

**Table:** 

Research suggests that the relative contribution of central and peripheral fatigue varies depending on exercise intensity. While psychological and central factors may predominate in moderate-intensity exercise, peripheral fatigue plays a more significant role in high-intensity exercise, although it may always be influenced by central mechanisms.
Both task failure and fatigue cannot be attributed to a single threshold or limiting factor. Performance regulation depends on the interaction of multiple physiological and psychological systems and is modulated by individual characteristics such as experience, effort tolerance, aerobic capacity, or cognitive and emotional differences.
There are several models that attempt to explain the phenomenon of task failure in endurance exercise. However, their explanations of the role of central mechanisms and their interplay with peripheral mechanisms do not seem to be supported by current knowledge from neuroscience and cognitive neuroscience, or by robust empirical evidence. Thus, the interaction between physiological, psychological, and contextual factors remains a subject of debate and further models are needed.

## Introduction

Endurance sports, such as cycling and running, require individuals to strategically pace their effort over time in long-duration events (e.g., a Tour de France stage or a marathon) to reach their goals without premature exhaustion. However, these efforts are not always steady state; they often include phases of significantly increased intensity leading to fatigue, and ultimately if exercise is continued, exhaustion. For instance, in cycling, a ‘domestique’ may set the pace of their team on a climb and suddenly must reduce their speed because they are unable to sustain the required power output any longer. These moments have been described using various terms, such as point of exhaustion, tolerance limit, point of fatigue, or task failure [1]. However, this phenomenon can occur under a wide range of conditions, such as different durations or intensities [2], with or without prior fatigue [3], in hot or temperate environments [4] or during training or competition. What unites all these situations is that the athlete can no longer sustain the required intensity to achieve the goal of the task, even if the intention to do so remains intact. Moreover, task failure is not only context dependent, but also highly individualized [5]. The causes of failure may vary significantly between individuals, owing to differences in physiology, mental resilience, experience, or pacing strategies; and even within the same athlete, depending on the task or situation [6]. Therefore, this concept is central to understanding endurance performance, as it highlights the complex interplay between physical and psychological limitations.

In high-performance sports, having the ability to delay task failure by even a few seconds can mean the difference between victory and defeat. However, despite being a crucial phenomenon in sports performance, the factors that determine this point are still not fully understood. Indeed, there is debate in the literature as to whether task failure is determined by peripheral [1] or central mechanisms [7], or a combination of the two [8]. Peripheral mechanisms refer to physiological processes that occur within the muscles and at the neuromuscular junction. That is, they are related to muscle fatigue including factors such as progressive metabolite accumulation and reductions in muscle excitability, which physically limit the ability to continue executing the required level of force to complete a given task [9]. However, there are situations where peripheral models of task failure might not be sufficient to fully understand this phenomenon [10]. Indeed, central factors, referring to processes within the central nervous system (CNS) that influence motor command and subjective psychological dimensions, such as perceived effort (rating of perceived exertion [RPE]), motivation, attentional focus, or experience, are likely to play an important role [11]. The relationship between peripheral and central factors is critical to understand how and why task failure occurs, and how it affects both performance and task failure in both submaximal and maximal activities [12–14].

To better understand task failure, it is essential to define fatigue, a concept that underlies most models attempting to explain this phenomenon. Following the framework proposed by Enoka and Duchateau [15], fatigue can be divided into performance fatigability, referring to the objectively measurable decline in the ability to produce force or power, and perceived fatigability, which relates to the subjective sensations that influence an individual’s willingness to continue. These components interact dynamically and are influenced by multiple factors, including the individual’s physiological state, environmental conditions, and prior exertion [16]. For instance, task failure may arise due to muscular depletion during a prolonged climb, or alternatively due to a lack of motivational drive before reaching physiological limits. Therefore, understanding task failure requires a multifactorial perspective that integrates both peripheral and central mechanisms, as well as the interplay between them. This narrative review aims to critically examine the mechanisms underlying endurance performance regulation, summarize key theoretical models where relevant, and emphasize gaps in knowledge that require further investigation in the context of task failure.

## Peripheral Mechanisms and Their Relationship with Task Failure

Peripheral fatigue, as already noted, refers here to the physiological limitations at the muscular level and the neuromuscular junction that compromise the ability to sustain a target intensity during prolonged exercise [17]. Key mechanisms include excitation–contraction coupling failure, the accumulation of metabolites, and disruptions in neuronal activation [18, 19]. These factors directly impact the muscle’s capacity to produce force and power, particularly in sustained submaximal exercise, although the perturbations that ultimately result in task failure during severe-intensity exercise remain unclear, and a comprehensive evaluation of the different factors contributing to performance fatigability is still needed [20].

A primary contributor to peripheral fatigue is the accumulation of metabolites, such as hydrogen ions (H^+^), which disrupt cellular homeostasis and impair both muscle contraction and neuromuscular function. Additionally, the depletion of energy substrates, such as glycogen, reduces metabolic efficiency, also accelerating the decline in physical performance [1].

### Cardiovascular Contributions to Task Failure

The cardiovascular system plays a pivotal role in peripheral fatigue and task failure by regulating oxygen delivery, nutrient supply, and metabolite clearance during endurance exercise. Limitations in cardiac output, stroke volume, or blood flow redistribution can impair muscle oxidative capacity, accelerating the accumulation of fatigue-inducing metabolites such as H⁺ and inorganic phosphate (Pi), particularly during high-intensity efforts involving large muscle masses [1, 21]. For example, reduced oxygen delivery exacerbates anaerobic metabolism and intramuscular acidosis, directly contributing to declines in contractile function and force output [22]. In prolonged endurance activities, chronic cardiovascular adaptations, such as increased left ventricular mass and enhanced vascular efficiency, may initially support performance, but acute stressors such as dehydration or thermal strain can lead to cardiovascular drift, elevating heart rate and reducing stroke volume, thereby precipitating task failure [23]. Furthermore, cardiovascular strain interacts with central mechanisms through group III/IV muscle afferents, which convey metabolic and hemodynamic feedback to the CNS, which may modulate central motor drive (CMD) [24, 25]. These interactions emphasize the cardiovascular system’s integral role in the multifactorial regulation of task failure in endurance sports.

### Contractile Function of Muscle

The contractile function of muscle is crucial for understanding task failure in endurance sports, as any alteration in the underlying mechanisms can directly contribute to the muscle’s inability to maintain the required force. During prolonged and intense exercise, the interaction between sarcolemma excitability, calcium (Ca^2+^) kinetics, and metabolite accumulation is a critical factor that affects the muscle’s ability to continue functioning optimally, contributing to task failure.

#### Peripheral Metabolites in Skeletal Muscle

The intensity of exercise plays a critical role in the rate of metabolite accumulation, being faster and more pronounced during high-intensity exercise compared with lower intensity activities [26]. At higher intensities, the increased reliance on anaerobic pathways for rapid adenosine triphosphate (ATP) production leads to a more rapid accumulation of byproducts such as Pi and H^+^, owing to the increased metabolic rate outpacing the body’s ability to clear or buffer them [27]. Additionally, this rapid ATP utilization can lead to the depletion of high-energy phosphates, including phosphocreatine (PCr) and ATP itself, which further compromises energy availability for muscle contraction. These metabolites and energy substrate depletions directly interfere with the muscle’s ability to maintain its contractile function. Specifically, the accumulation of Pi, a byproduct of ATP and PCr degradation during muscle contraction, reduces the sensitivity of muscle fibers to Ca^2+^, altering the excitation–contraction coupling mechanism [12]. This accumulation, combined with low PCr and ATP levels, ultimately leads to a reduction in the force generated by muscle fibers, limiting their capacity to maintain sustained contractions [12].

The presence of H^+^ ions increases intracellular acidosis, leading to a decrease in pH within muscle fibers. Hearris et al. [28] emphasize that this reduction in pH compromises sarcolemma excitability, and also directly interferes with the contractile elements, affecting the transmission of action potentials and, therefore, the muscle’s ability to generate effective contractions. This intracellular acidosis is especially relevant during high-intensity exercise, where the accumulation of H^+^ is greater and precipitates task failure by reducing the muscle’s response to nerve stimuli. In contrast, during lower intensity exercise, the acid–base balance can be maintained for longer periods because the primary energy system used is aerobic metabolism, which produces fewer acidifying byproducts. Therefore, the body’s buffering systems can effectively neutralize the smaller amounts of H^+^ generated [22, 29].

The accumulation of H^+^ challenges the muscle’s buffering systems by exceeding their capacity to maintain pH homeostasis, while Pi primarily interferes with Ca^2+^ release and muscle contractile sensitivity. This imbalance leads to an inability to effectively neutralize the increase in acidity, contributing to muscle fatigue. Additionally, Pi interferes with Ca^2+^ release and sensitivity in the muscle, further impairing muscle contraction [30]. Furthermore, the depletion of PCr, a key buffer for ATP resynthesis, exacerbates these effects by limiting rapid energy replenishment during intense efforts. The depletion of glycogen stores during prolonged exercise reduces the muscle’s ability to sustain high-intensity efforts, as glycogen is a key substrate for energy production during these activities [31, 32]. In a glycogen-depleted state, the muscle shifts to alternative energy sources, such as fats and amino acids, which are less efficient at supporting high-intensity exercise and lead to a decrease in power output [33, 34]. This shift is compounded by reduced PCr availability, which impairs the muscle’s ability to buffer ATP declines and maintain performance. Furthermore, as glycogen availability decreases, the muscle’s capacity to produce lactate (La^–^) also diminishes, affecting both the absolute level of La^–^ production and the La^–^ threshold during high-intensity efforts [35]. This shift in energy metabolism particularly impairs endurance performance [36], such as time-to-exhaustion tests or time-trial performance, which require a sustained power output over long durations. Simultaneously, the increase in H^+^ in muscle tissue and blood reduced the pH, further inhibiting enzyme activity and disrupting the contractile proteins’ function. This combination of metabolic strain and pH imbalance, along with ATP and PCr depletion, compromises both energy production and contractile function, leading to a decline in muscle performance and increasing the likelihood of task failure [37].

Although there are mechanisms that temporarily counteract the effects of the accumulation of these metabolites, such as the removal of H^+^ through the blood to maintain the acid–base balance [38], these mechanisms may not be sufficient when the rate of H^+^ production from ATP resynthesis, driven by exercise intensity, exceeds the muscles’ buffering capacity. Similarly, PCr serves as an important buffer for ATP, but its depletion during high-intensity exercise can overwhelm these compensatory systems. In lower intensity exercise, the buffering capacity is typically sufficient to maintain the acid–base balance without being overwhelmed, allowing the muscle to continue functioning properly for a longer time.

In summary, the accumulation of Pi and H^+^, alongside the depletion of PCr and ATP, acts as a critical limiting factor in the muscle’s ability to maintain contraction during prolonged exercise. Their impact on excitation–contraction coupling and the reduction in muscle excitability are determinants in the onset of task failure, highlighting the importance of these processes in regulating performance in endurance sports.

##### Sarcolemma Excitability

Sarcolemma excitation is a fundamental process for the propagation of action potentials that allow muscle contraction. This process depends on the proper regulation of sodium (Na^+^), potassium (K^+^), and chloride (Cl^−^) channels, which maintain the action potential necessary for muscle fibers to contract effectively [39, 40]. During muscle contraction, these channels facilitate the depolarization and repolarization of the membrane, allowing the release of Ca^2+^ from the sarcoplasmic reticulum into the sarcoplasm, which activates the myofilaments for contraction [41].

During prolonged exercise, there are alterations in ionic balance, including the loss of K^+^ from muscle fibers into the extracellular space. This loss of K^+^ is mainly due to increased depolarization of the membrane and disruption of normal ionic gradients during muscle contraction. As a result, the concentration of K^+^ in the extracellular space increases, which can significantly impact muscle excitability. This process is exacerbated by the size of the muscle groups involved and the intensity of the exercise. Additionally, the reduction in the K^+^ gradient affects the activity of ion channels, particularly the Cl^–^ channels, such as ClC-1, which play a crucial role in maintaining sarcolemmal excitability. The decrease in the function of these channels impairs the ability of muscle fibers to maintain action potentials [42]. These ionic alterations limit the muscle’s ability to generate action potentials and ultimately reduce its contraction capacity [43]. Therefore, the reduction in sarcolemmal excitability leads to impaired force generation during repeated contractions, as muscle fibers struggle to propagate effective action potentials. This progressive decline in muscle force output directly impacts performance, particularly during high-intensity or prolonged tasks, where the inability to maintain sufficient excitability contributes to task failure.

The intensity of exercise plays a critical role in how these ionic disturbances affect muscle function. In low-intensity exercise, the metabolic demand on muscle fibers is lower, resulting in reduced activation frequency and less frequent depolarization events. Consequently, the ionic pumps, such as the Na^+^/K^+^ ATPase, can effectively restore ionic gradients, and the muscle fibers can maintain their excitability for longer periods, as the rate of K^+^ loss and the subsequent impact on muscle excitability are much slower [42]. However, when exercise intensity is higher, these ionic changes accumulate more rapidly, and the ability of muscle fibers to maintain action potentials diminishes at a faster rate. This decrease in contraction efficiency leads to a higher energy expenditure, as muscles recruit a greater percentage of fibers and increase ATP utilization to maintain the required intensity [44]. Additionally, the alteration of ionic gradients and K^+^ loss contribute to a higher oxygen cost (VO_2_) due to the increased activity of ion pumps, such as the Na⁺/K⁺ ATPase, which work to restore homeostasis during muscle contractions. This process requires additional ATP, thereby elevating metabolic demand and VO_2_ [45]. These factors collectively limit the athlete’s ability to maintain submaximal or maximal intensities and are key in the onset of task failure in endurance sports, where peripheral changes directly affect the muscle’s ability to respond to physical demand [46, 47].

Grosicki et al. [47] emphasize that the performance of fast-twitch fibers (major histocompatibility complex IIa) plays a critical role in maintaining force production during maximal efforts. These fibers are particularly vulnerable to a decline in excitability under prolonged or intense demands, which is especially relevant during critical phases of competition. The inability of these fast fibers to sustain performance during decisive moments can directly precipitate task failure, particularly in events requiring a sustained final effort. This phenomenon is closely tied to the muscles’ capacity to maintain ionic homeostasis during exercise.

The function of ATP-sensitive potassium channels (KATP) and ClC-1 channels is essential to mitigate the effects of extracellular K^+^ accumulation and ensure efficient excitation–contraction coupling. However, as Renaud et al. [43] showed the effectiveness of these channels in maintaining ionic balance diminishes under prolonged stress. This reduction impacts Ca^2+^ release from the sarcoplasmic reticulum, weakening contractile force and ultimately contributing to task failure [17]. Rather than being a gradual process, this breakdown in physiological mechanisms can reach a critical threshold where the muscles are no longer able to generate the required force, marking the point of task failure.

##### Calcium Kinetics

While prolonged exercise may lead to an accumulation of metabolites, such as Pi and magnesium ions (Mg^2+^), which impair the sarcoplasmic reticulum’s ability to release and reuptake Ca^2+^ [48], it is important to distinguish these cumulative effects from the mechanisms directly leading to task failure. Task failure can sometimes appear to occur independently of the gradual onset of fatigue, highlighting the need to differentiate between acute performance limitations and progressive fatigue-related impairments. Grosicki et al. [47] emphasize that sustained effort may reduce the effectiveness of Ca^2+^ channels, limiting the availability of Ca^2+^ for contraction and impacting force production. However, this process primarily reflects long-term reductions in performance capacity rather than an immediate determinant of task failure.

During exercise, Ca^2+^ release and reuptake dynamics in the sarcoplasmic reticulum adapt based on the intensity and duration. At low intensities, the slower rates of Ca^2+^ cycling allow the sarcoplasmic reticulum to regulate Ca^2+^ effectively over time [49]. However, prolonged low-intensity efforts gradually impair Ca^2+^ handling efficiency, albeit more slowly than high-intensity efforts. As exercise intensity increases, the recruitment of fast-twitch muscle fibers becomes critical for generating rapid and powerful contractions, heavily reliant on efficient Ca^2+^ release. The high rate of Ca^2+^ flux during intense exercise places significant demands on Ca^2+^ pumps, increasing the risk of functional overload and compromising Ca^2+^ gradients [50]. This, coupled with the accumulation of metabolites such as Pi and Mg^2+^, inhibits Ca^2+^ release from the sarcoplasmic reticulum, disproportionately affecting fast-twitch fibers under metabolic and mechanical stress [50]. Prolonged or intense exertion exacerbates these inefficiencies owing to changes in ryanodine receptor channels, responsible for Ca^2+^ release during contraction. Oxidative stress and elevated cytosolic Ca^2+^ levels can promote the dissociation of stabilizing proteins such as calstabin from the ryanodine receptor complex, leading to Ca^2+^ leaks from the sarcoplasmic reticulum [51, 52]. This reduces the Ca^2+^ available for subsequent contractions, impairing force generation and increasing the likelihood of task failure [47]. These effects are particularly pronounced during high-intensity or maximum-effort scenarios, where precise Ca^2+^ cycling is essential to sustain muscle output [50].

In addition to Ca^2+^ release, the structure of the T-tubules plays a key role in transmitting the action potential into the muscle fiber. Caldwell et al. [53] highlight that, during prolonged exercise, the integrity of the T-tubule membrane and associated ion channels may deteriorate, affecting the efficiency of nerve impulse transmission [30]. This deterioration can disrupt the balance of essential ions such as Na^+^ and K^+^, which are critical for proper excitation–contraction coupling. As a result, the muscle’s ability to respond to exercise demands is further reduced. Coyle and Edward [54] suggest that the disruption of this mechanism is one of the main reasons muscles fail to generate the necessary force to maintain exercise intensity, contributing to task failure.

In summary, the deterioration of Ca^2+^ kinetics and structural alterations directly compromise the muscle’s ability to sustain contraction. This decline is particularly critical during prolonged or intense efforts, where efficient Ca^2+^ handling is essential to maintain force production. These findings highlight the role of Ca^2+^ dynamics in determining muscle performance and the onset of task failure, particularly in endurance events where sustained contraction is required.

### Neuromuscular Activation

Neuromuscular activation refers to the process by which the CNS controls the activity of muscle fibers, allowing the generation of force in response to external stimuli [55]. In the context of task failure, the CNS’s ability to optimally activate muscle fibers plays a critical role. A decline in this capacity can compromise motor performance, reducing the force generated and accelerating the progression toward task failure. This decline involves processes such as decreased activation, altered functioning of spinal motoneurons, and modifications in afferent feedback, all of which interact to impair the CNS’s capacity to sustain effective neural drive to the muscles [56].

During long-duration exercise, the CNS becomes less effective at maintaining neural drive to the muscles [57, 58]. This reduction in CNS function is thought to be caused by several factors, including an increase in brain serotonin levels, which can reduce motor neuron excitability, and a depletion of neurotransmitters such as dopamine that are essential for motor control [59]. Additionally, the accumulation of metabolites such as ammonia, as well as altered ion concentrations, can interfere with the brain’s ability to generate effective motor commands [60], which translate into a decreased capacity of motor units to generate force [18]. Research by Torres-Peralta et al. [61] showed that, near task failure, muscle activation is significantly reduced, suggesting that central mechanisms play an important role in limiting performance. This decrease in neural activation is likely to be a key factor contributing to athletes’ inability to sustain exercise intensity, particularly in endurance sports [62].

Spinal motoneurons, which relay signals from the CNS to muscle fibers, are influenced by both central and peripheral fatigue. Central fatigue reduces the descending motor drive to the spinal motoneurons, while peripheral fatigue impairs their excitability, limiting the muscles’ response to motor commands. Spinal motoneurons, which transmit signals from the CNS to muscle fibers, are also affected during prolonged exercise. The development of both central and peripheral fatigue can alter the efficiency of this spinal transmission, limiting the muscles’ ability to respond to signals sent from the brain. Eklund et al. [63] indicate that as intense exercise progresses, the efficiency of motoneurons in transmitting impulses to muscles decreases owing to mechanisms such as increased inhibitory feedback from muscle afferents (groups III and IV) and altered excitability of motoneurons, both of which limit the neural drive required for effective muscle activation. This reduction in efficiency is partly due to neural adaptations and changes in activation, including decreased recruitment of high-threshold motor units and reduced firing rates of motoneurons [63]. These changes are influenced by increased inhibitory input from muscle afferents (groups III and IV), which respond to the accumulation of metabolites such as La^–^ and H^+^ [64]. In addition, central mechanisms, such as diminished corticospinal excitability and impaired synaptic transmission, further reduce the motor output, collectively affecting the muscle’s ability to sustain force production [65]. This deterioration in neuromuscular function contributes to reduced athletic performance, bringing the athlete closer to task failure by compromising the consistent activation of motoneurons required for sustained force output. If exercise intensity is sustained, this reduced motoneuron efficiency acts as a contributing factor by limiting motor unit recruitment and force production, especially under high-demand conditions, ultimately leading to task failure.

Overall, neuromuscular activation directly influences the muscles’ ability to sustain contraction during prolonged exercise. The reduction in activation, the decreased efficiency of spinal motoneurons, and the decline in afferent feedback all compromise the athlete’s ability to maintain effort, making task failure inevitable when physical demand exceeds the nervous system’s capacity to maintain muscle activation.

### Critical Threshold of Peripheral Fatigue

As discussed in the previous section, the accumulation of metabolites, including Pi and H^+^, within the muscle during high-intensity exercise leads to a reduction in the muscle fibers’ ability to sustain effective contractions [21, 46]. Evidence suggests that these metabolites act as feedback signals to the CNS through group III and IV afferents, exerting inhibitory feedback on CMD and thereby reducing muscular force output to preserve homeostasis [24]. For instance, in the study conducted by Amann et al. [24], peripheral fatigue was assessed through changes in quadriceps force in response to supramaximal magnetic femoral nerve stimulation before and after constant load cycling to exhaustion at 80% peak power output. The results showed that quadriceps force dropped to a critical level, after which participants were unable to maintain the required effort. This suggests there is a limit to how much force reduction participants can tolerate before task failure occurs. Fatigue was quantified by measuring the decrease in quadriceps twitch force from before to after exercise. Meanwhile, Hureau et al. [66] reported that during repeated sprints, performance seems modulated to ensure that peripheral fatigue does not exceed a critical threshold, regardless of the level of pre-existing fatigue. This regulation would be mediated by afferent feedback from group III to IV muscle afferents, providing the CNS with real-time information about metabolic and mechanical changes within the muscle. Specifically, these afferents respond to intramuscular accumulation of metabolites such as Pi, K^+^, and H^+^, as well as reductions in intracellular pH. The CNS would integrate this feedback to adjust CMD, reducing the power output to prevent further deterioration in muscle contractile function. This protective mechanism likely involves inhibitory signals from the muscle afferents that limit motor unit recruitment and prevent peripheral fatigue from surpassing a critical sensory threshold, thereby safeguarding muscle function during high-intensity exercise. This critical threshold refers to the level of peripheral fatigue at which performance is significantly compromised, and adjustments in power output during the sprints are aimed at preventing this threshold from being surpassed. These mechanisms appear to be a strategy of the body to prevent complete exhaustion of the muscles and potential physiological damage, suggesting that task failure may occur as a protective mechanism for the body [7].

Amann [46] proposes that peripheral fatigue progresses until reaching an individual critical threshold, beyond which the body regulates exercise intensity to prevent excessive deterioration of muscle function. This process would involve both the neuromuscular system and CNS, which work together to regulate muscle activation and reduce force production when the critical threshold is reached. Consequently, the body responds by modulating CMD, leading to a reduction in exercise intensity to preserve homeostasis and prevent excessive peripheral fatigue. In extreme cases, when the critical threshold of fatigue is surpassed, this regulatory mechanism results in the cessation of physical activity to protect the integrity of neuromuscular function and prevent irreversible damage to muscle tissue and other physiological systems, such as cardiovascular and respiratory functions, thus maintaining homeostasis during exercise. This suggests that task failure is not solely a matter of muscle exhaustion, but also a protective mechanism aimed at safeguarding the body from further physiological harm [66].

However, recent research has questioned whether peripheral fatigue is strictly regulated by a critical threshold. For example, Thomas et al. [21] argued that peripheral fatigue is not strictly regulated by a specific threshold but is instead influenced by the mode and intensity of exercise, as well as by physiological adjustments across various systems, including the cardiovascular and respiratory systems. Their evidence is drawn from studies showing that the reduction in skeletal muscle contractile function, quantified as the decline in potentiated twitch force following exercise, varies depending on the active muscle mass and exercise modality. Specifically, when a smaller muscle mass is engaged, greater reductions in contractile function can be tolerated before fatigue becomes intolerable, as the sensory feedback is confined to a smaller muscle mass, and disruptions to other physiological systems are minimal. In contrast, when a larger muscle mass is involved, the demands placed on this larger mass, along with the greater strain on other physiological systems, combine to modulate the development of fatigue [21]. Consequently, the degree of fatigue that can be tolerated is mediated not only by adjustments in the muscle itself, but also by compensatory adjustments in other physiological systems. This suggests that peripheral fatigue is not governed by a single critical threshold, but rather by a complex interplay of factors, including muscle mass, exercise intensity, and the body’s ability to maintain homeostasis during physical exertion. Additionally, Black et al. [67] investigated the muscle metabolic and neuromuscular responses to exercise across different intensity domains. They found that during severe-intensity cycling (above critical power [CP]), task failure was consistently associated with a muscle metabolic milieu characterized by low pH, depleted PCr, and elevated La^–^. However, this did not appear to be directly linked to central regulation of motor output. In contrast, during moderate-intensity and heavy-intensity exercise (< CP), the neuromuscular and metabolic patterns differed, suggesting that the determinants of fatigue vary by intensity. These findings support the notion that task failure emerges from intensity-dependent interactions between metabolic disturbance, neuromuscular function, and possibly central mechanisms, rather than being governed by a fixed critical threshold.

Peripheral fatigue and the concept of a critical threshold of fatigue have been proposed as important mechanisms in regulating physical performance during prolonged exercise [25, 67, 68]. The interaction between afferent signals from fatigued muscles and the CNS plays a role in regulating exercise intensity to prevent irreversible muscle function deterioration. This process, which may act as a protective mechanism, is related to the occurrence of task failure. However, the role of the critical threshold as a determining factor is not entirely clear and remains a subject of debate. In some contexts, when peripheral fatigue reaches a critical level, the body adjusts exercise intensity or even forces the athlete to stop, leading to task failure. Nonetheless, recent literature suggests that peripheral fatigue does not necessarily follow this specific threshold pattern, and factors such as exercise type, intensity, and the individual characteristics and context of athletes can significantly influence how fatigue is regulated [1, 21, 69]. Therefore, while the concept of a critical threshold holds significant relevance, its association with peripheral fatigue is more established. However, its potential role as a central mechanism in performance regulation remains to be fully clarified.

## Central Mechanisms and Their Relationship with Task Failure: Physiology

In this section, we critically review how central processes could influence an athlete’s decision to either continue or stop exercising. For decades, researchers have sought to identify central physiological mechanisms that constrain endurance performance, using approaches that range from measurements of neurotransmitters to electroencephalography (EEG), functional neuroimaging, and non-invasive brain stimulation. Despite this long-standing interest, direct empirical evidence captured during exercise, particularly at the point of task failure, remains scarce and methodologically limited.

### Metabolites and Neurotransmitters

Just as skeletal muscles alter their function during exercise [70], the same would presumably occur at the brain level [71]. For instance, the role of neurotransmitters and metabolites would be fundamental in regulating performance during prolonged exercise, particularly in the onset of task failure. During endurance exercise, the balance between neurotransmitters such as serotonin, dopamine, and norepinephrine seems to play a crucial role in the ability of the CNS to sustain motor activation [72, 73], suggesting a plausible role for these mechanisms, but definitive human evidence, especially at the point of task failure, remains to be established.

Animal studies synthesized in the review by Meeusen et al. [74] indicate that prolonged running can alter serotonin levels in motor-related brain regions, including the hypothalamus and raphe nuclei, but the direction and magnitude of these changes are not uniform. Increases in central serotonin have been associated with higher plasma-free tryptophan, enhanced uptake into the brain, and upregulation of tryptophan hydroxylase activity, potentially leading to increased turnover [75–77]. In parallel, evidence on dopamine from animal studies is even more mixed: while some experiments show a reduced dopamine turnover and release in striatal and hypothalamic regions under conditions of prolonged or high-intensity exercise, others report no changes or even increases depending on the brain region and the methodology used [74, 78, 79]. Peripheral metabolic changes, including increased free fatty acids and altered branched-chain amino acid flux, have been proposed to indirectly impair dopamine synthesis and release, which could reinforce dopaminergic alterations seen in animal models.

Beyond serotonin and dopamine, animal studies have also reported exercise-induced changes in other neurotransmitters and neuromodulators, including noradrenaline, adrenaline, and glutamate, which may be integral to cortical excitability, motor control, and autonomic regulation during prolonged exertion [74, 78]. Classic microdialysis work in rodents showed that adrenaline is released as a neurotransmitter in the hypothalamus and that its release can be selectively modulated by brainstem stimulation [80], reinforcing the idea of a broader catecholaminergic and excitatory amino acid involvement during sustained effort, although findings across studies remain heterogeneous.

In humans, direct in-vivo measurement of these central neurotransmitters during exercise is not feasible for ethical and technical reasons [74, 78]. Consequently, evidence remains indirect and derives from proxies: increases in prolactin as a serotonergic marker during prolonged submaximal exercise [81]; plasma catecholamines or peripheral markers of glutamatergic turnover; and positron emission tomography or brain-stimulation paradigms reporting exercise-related dopaminergic changes. More recently, an exploratory study using proton magnetic resonance spectroscopy reported ≈11% higher glutamate and ≈12% higher glutamate + glutamine signals in the occipito-parietal cortex immediately after maximal graded exercise in healthy young male individuals, alongside elevated brain La^–^, although the physiological mechanisms and their relevance to task failure remain uncertain [82]. A systematic review [83] synthesizing human studies concluded that, although some studies show exercise-related changes in dopaminergic and serotonergic markers, the evidence base is highly heterogeneous and predominantly of low methodological quality, which precludes a meta-analysis and calls for caution when extrapolating to mechanisms of task failure in humans.

Alongside these central neurotransmitter shifts, peripheral metabolite accumulation (H⁺, Pi) during prolonged or high-intensity exercise may depress muscle contractility and increase group III/IV afferent discharge, intensifying ascending signals to the CNS [84]. Concurrently, shifts in tryptophan metabolism have been proposed based on animal models, whereby non-esterified fatty acids displace tryptophan from albumin, increasing free tryptophan availability for brain uptake and favoring 5-HT synthesis; most tryptophan is then metabolized along the kynurenine pathway, producing neuroactive metabolites [75, 77]. In trained athletes, exhaustive aerobic exercise has been reported to modulate this peripheral tryptophan-kynurenine axis during or immediately after exercise, suggesting an indirect peripheral-central link [85]. In sum, although there is agreement on the relevance of neurotransmitters and peripheral metabolite accumulation in endurance performance in general, and task failure in particular, the available empirical evidence does not allow the drawing of any firm conclusions.

### Neural Activity During Exercise

Direct measurements of brain activity during exercise are scarce, and, to the best of our knowledge, there is no empirical published evidence specifically captured at the point of task failure. Most human studies have used EEG or functional near-infrared spectroscopy (fNIRS) to monitor neural dynamics, whereas functional magnetic resonance imaging protocols addressing exercise typically employ low-to-moderate intensities in highly constrained (and therefore less ecological) environments. Reviews of the literature [e.g., 86, cf. 87] highlight that, although these techniques can provide useful information about global electrical or hemodynamic responses to exercise, they offer limited spatial specificity and very little direct evidence concerning the neural events that precipitate task failure in ecologically valid, high-intensity settings.

Electroencephalography provides excellent temporal resolution and can index changes in cortical oscillatory activity and event-related potentials during exercise. However, at moderate-to-high intensities, EEG faces substantial methodological challenges: movement and muscular artifacts (including muscle noise contamination), large cardiorespiratory and sweating-related noise, and changes in electrode impedance. These issues escalate with exercise intensity and complicate the interpretation of signals recorded close to exhaustion. Consequently, while some EEG studies report modulation of neural oscillations during sustained exercise [e.g., 88], to the best of our knowledge, there are no published studies measuring EEG immediately before and at task failure. In addition, changes in corticospinal and motoneuronal excitability have been observed during fatiguing exercise, suggesting that the corticomotoneuronal pathway itself may contribute to central fatigue and be a target for endogenous or exogenous neuromodulation [89].

The fNIRS technique is attractive for field-compatible recordings because it tolerates more movement than functional magnetic resonance imaging and can be deployed during cycling or running. Yet, standard fNIRS approaches mainly capture superficial (frontal) hemodynamic changes and are highly susceptible to systemic confounds (e.g., changes in scalp blood flow, blood pressure, and skin perfusion). Thus, positive fNIRS findings, often reported in prefrontal regions, should be interpreted cautiously: they point to changes in cortical hemodynamics during exercise but do not allow precise localization of the deeper neural substrates or unambiguous attribution to cognitive versus peripheral physiological drivers [90]. Many fNIRS studies have been conducted at submaximal intensities and are limited in sample size, which again constrains inference about neural activity at task failure [87].

Functional magnetic resonance imaging and magnetic resonance spectroscopy offer spatial precision and biochemical indices, but their application to exercise research is constrained by practical and ecological problems: participants are supine, exercise intensities are low to moderate, and movement is minimized, which limits generalizability to real-world high-intensity exertion and exhaustion. Therefore, functional magnetic resonance imaging/magnetic resonance spectroscopy results provide complementary information on brain systems involved in sustained attention, reward, or autonomic regulation under low-intensity exercise, but they are of limited value for explaining neural dynamics at true task failure.

Perturbation approaches, such as transcranial direct current stimulation and transcranial alternating current stimulation, have been used experimentally to probe causal contributions of cortical areas to endurance and perceived exertion (RPE) [89]. However, movement-related artifacts, a low signal-to-noise ratio at high intensities, confounding peripheral physiological signals (cardiac and respiratory), and the generally small sample sizes and heterogeneity of protocols all reduce confidence in existing findings [91]. Indeed, a recent umbrella meta-analysis points to small and inconsistent effects of non-invasive stimulation on performance and RPE [92], with potential small-sample and publication biases. Hence, to date, this research provides little knowledge regarding mechanistic evidence for the brain regions or processes that trigger task failure.

Taken together, the neurophysiological literature suggests that while the brain is clearly engaged during prolonged exercise, current direct evidence about neural dynamics at task failure is minimal and methodologically limited. For this reason, claims about specific cortical loci or definitive neural mechanisms underlying the decision to stop remain highly speculative and must be tempered.

## Central Mechanisms and Their Relationship with Task Failure: Psychology

Central mechanisms linked to endurance, in contrast to peripheral mechanisms, encompass both physiological and psychological components. While the preceding sections have emphasized the limited and indirect evidence regarding central physiological processes, there is also a parallel and equally important line of inquiry into psychological mechanisms. Research has focused on the perception of effort, perceived exertion, and affective–emotional responses as key constructs to understand how athletes interpret and regulate prolonged exertion.^1^

Rating of perceived exertion represents the subjective dimension of physical exercise that has been most extensively studied. Across a wide range of tasks, ratings of RPE consistently rise with exercise intensity and duration, even when peripheral markers of fatigue remain relatively stable [93, 94]. Steele [94] expands on this perspective by highlighting that RPE not only indexes physical demand but also informs self-regulation and strategic decision making about when to stop exercise, influenced by previous experience, training, or personal expectations. This author further argues that RPE can act as a protective mechanism, preventing physical damage by inducing a conscious sense of exhaustion and motivating the individual to stop before reaching dangerously high levels of fatigue.

According to some theoretical accounts [e.g., 95], RPE, rather than peripheral afferent feedback, is the primary determinant of exercise termination. To test this hypothesis, many studies have used mental-fatigue paradigms [96], reasoning that pre-exercise cognitive load should elevate RPE and thereby reduce endurance performance [97].

However, this literature has been questioned recently. For instance, Holgado et al. [98] failed to replicate the findings of one of the most cited articles on this topic [97]. Subsequent reviews and meta-analyses reported mixed or null effects [13, 99]. A more recent work concluded that the available studies are underpowered because of small sample sizes and exhibit signs of publication bias [92]. When correcting for this bias, the reported effects on endurance performance and RPE become negligible, and Bayesian analyses show no credible evidence supporting a true effect. Moreover, prolonged cognitive tasks, such as those used in these studies (e.g., Stroop task) may induce other subjective experiences, such as boredom or attentional shifts [100], over and above any mental fatigue, which further complicate any interpretation of findings from this literature.

Beyond RPE, the affective dimension of exercise has also been intensively investigated. According to Ekkekakis’s dual-mode model, affective valence becomes increasingly negative at higher exercise intensities, which may interact with RPE to influence the decision to stop [101, 102]. Affective valence, or the positive or negative emotional experience during exercise, can modulate an athlete’s ability to sustain effort. Ekkekakis [101, 102] suggests that a positive affective state during exercise may be associated with greater tolerance to exertion and a lower perception of fatigue, while negative emotions may accelerate the onset of task failure. For instance, recent research by Farias-Junior et al. [103] found that affective valence during low-volume high-intensity interval exercise declines significantly as the session progresses, accompanied by an increase in RPE and heart rate. In their study, physical activity level and RPE were identified as primary predictors of in-task affective valence across different phases of the training session. This suggests that individual differences, such as physical activity level and the RPE, play a crucial role in how athletes emotionally experience high-intensity interval training, and consequently affect their ability to sustain effort and manage the psychological demands of exercise as intensity fluctuates.

While the literature has primarily focused on RPE and affective valence, it has often overlooked the broader richness of subjective experience during exercise [6, 104]. For instance, emerging evidence suggests that states of flow or optimal experience can enhance persistence and performance [105], but these states may be disrupted or absent as athletes approach task failure.

However, the current evidence base remains sparse and fragmented. There is still a clear need for more systematic research examining subjective experience beyond ratings of RPE to understand task failure in a more complete way. Although athletes’ subjective experiences of exertion and task failure can vary widely [104], most studies aggregate data at the group level and very few attempt to draw conclusions at the individual level [cf. 106]. Recent work has begun to address this gap. A large-scale survey of licensed cyclists documented substantial variability in how riders report reaching their limit, highlighting differences in RPE, bodily sensations, and the perceived role of motivation [104]. Complementary qualitative interviews [6] explored in depth how professional and amateur cyclists experience task failure during high-intensity efforts, revealing themes such as temporal distortion, bodily sensations, and the perceived interplay between “body” and “mind” at the point of task failure [6]. In parallel, an experimental study using the temporal experience tracing (see [107–109] for details on this novel methodology to dynamically track people’s subjective experience) method captured minute-by-minute fluctuations in five subjective dimensions during a 30-min dual-task cycling protocol [110]. This approach showed that physical effort tracked power output dynamically, while other dimensions (pleasure, boredom, mind wandering) displayed individual and time-dependent patterns largely uncaptured by conventional RPE measures. Additionally, a recent pre-registered experiment [111] involving trained cyclists examined whether access to real-time power data influenced performance and perceived voluntariness of task failure; the results showed higher maximal aerobic power when power feedback was available but minimal changes in maximal oxygen uptake, heart rate, or RPE, and only a modest order-dependent effect on perceived voluntariness, highlighting that contextual factors can subtly shape subjective experience at task failure. Together, these findings underscore the need for more systematic, fine-grained, and individualized approaches to studying subjective experience during exercise and task failure. Moving beyond single-point RPE scores toward repeated or mixed-method designs can help disentangle the interplay between RPE, affective responses, and motivational drive, and identify whether changes in subjective experience precede or follow declines in performance.

## Discussing Models of Endurance Performance Related to Task Failure

A person’s ability to sustain prolonged exercise is based on the integration of multiple signals from both the brain and body, meaning that factors such as motivation, RPE, and affective valence are key to an athlete’s ability performance. Over time, various authors have proposed different models to explain the factors contributing to task failure during exercise [e.g., 1, 7, 8, 10, 97].

Some of the work in this area was published by Noakes et al. [7], who proposed the “central governor” model of exercise performance. This model suggests that the brain uses fatigue symptoms as regulatory mechanisms to prevent physiological damage, emphasizing that task failure is ultimately determined by central control rather than peripheral limitations. While historically influential, this theory has been increasingly criticized for its lack of empirical support and its reliance on the outdated notion of a single “governor” sending inhibitory signals [95, 112]. Contemporary neuroscience instead points towards distributed and integrated brain processes, emphasizing concepts such as brain degeneracy (i.e., the capacity of different neural structures to produce the same output or function) [113], and highlighting the risks of reverse inference when linking neural activity to behavior [114].

In parallel with the central governor model by Noakes et al., Marcora et al. [97] proposed their psychobiological model of performance, which argues that exercise cessation is the result of conscious voluntary processes, with RPE as the main determinant, rather than the afferent signals (e.g., muscle, cardiovascular). While this model introduced important psychological constructs such as RPE and motivation, its assumptions have been increasingly challenged. As discussed above, to test the role of psychological factors in exercise performance, the psychobiological framework assumes that a prior cognitive task induces mental fatigue, which in turn elevates RPE and consequently reduces physical performance. However, recent reviews have questioned each step of this chain of reasoning: the cognitive tasks used in many studies may not reliably induce mental fatigue per se, or may produce other subjective states (e.g. reduced motivation, boredom) with potentially a greater impact on performance. Moreover, the evidence that prior mental/cognitive load (i.e., as a result of performing a cognitive task) consistently increases RPE and diminishes objective performance does not seem to be supported by the empirical evidence [115]. Furthermore, the idea that exercise cessation is purely a conscious decision oversimplifies the complex interplay between central and peripheral mechanisms [6, 104, 111], which likely operate beyond full conscious awareness. Moreover, it neglects all the literature on the long-standing issue of free will and conscious decision making [116, 117].

Burnley and Jones [1] acknowledge that both central and peripheral fatigue contribute to task failure, but suggest the relative contribution is differentiated by the domain of the exercise intensity. In moderate-intensity exercise, psychological aspects and central fatigue would be most relevant, while with severe-intensity exercise (above CP), it is suggested that the phenomenon is predominantly physiological with little influence of internal or external motivation on exercise tolerance or time to task failure [1]. This is supported by studies demonstrating that exercise maintenance above CP largely depends on muscle capacity to manage metabolite accumulation and PCr depletion [12, 28, 29, 118]. However, some cognitive manipulation studies [119–121] appear to contradict this view. These studies extended the time to exhaustion through psychological interventions, such as manipulating attentional focus and providing motivational cues [121]. Presumably, these techniques would help participants shift their perception away from discomfort, thereby reducing the RPE required to sustain the exercise.

More recently, Iannetta et al. [8] have highlighted the influence of perceptual factors, such as the RPE and muscle pain in determining factors for task failure. Their study shows that at the limit of tolerance, RPE reaches maximal or near-maximal levels across all exercise intensity domains, suggesting a central role of RPE in determining task failure. Furthermore, they observed domain-specific differences in perceptual responses: while global fatigue ratings increased with lower exercise intensities, sensations such as pain and breathlessness were more pronounced in higher intensity domains. These findings indicate that perceptual responses, shaped by both central and peripheral mechanisms, integrate to influence the decision to terminate exercise. For example, in Iannetta et al.’s study [8], central fatigue was present after moderate-intensity, heavy-intensity, and severe-intensity exercise but absent following extreme-intensity exercise, where peripheral fatigue was dominant. These domain-specific dynamics highlight the interplay between perceptual and neuromuscular factors in modulating task failure, supporting the idea that fatigue results from a complex interaction of physiological and perceptual responses rather than a single limiting factor.

Based on the multifactorial fatigue model proposed by Thomas et al. [21], task failure is suggested to occur when the cumulative effect of various inhibitory afferents generated by body regions involved in the exercise task reaches intolerable levels. It posits that, rather than being driven by a single threshold, as other authors proposed [46, 122], the regulation of fatigability depends on the exercise mode, intensity, and interactions between various physiological systems, including the cardiovascular, respiratory, and muscular systems [21]. Factors such as aerobic capacity, age, training history, and individual differences in fatigue tolerance must also be considered, as each individual responds uniquely to exercise [104], implying that models aimed at explaining endurance performance and task failure should be tailored to individual characteristics. For example, some athletes may have a higher tolerance for physical effort because of better recovery capabilities, while others may experience earlier fatigue owing to their physiological or psychological response [123, 124].

## Conclusions

The phenomenon of task failure in endurance sports would arise from the interaction of physiological and psychological factors. Task failure is a multifactorial phenomenon that cannot be explained purely by physiological mechanisms, as psychological factors are also involved. For instance, the perception of effort and exertion may play a crucial role in an athlete’s decision to continue or stop. This review also highlights that the subjective experience goes far beyond what can be captured by RPE and encompasses a wide range of phenomenological experiences that may also have a key influence in task failure [110].

Moving forward, if the field aims to provide an up-to-date and critically informed understanding of task failure, it must explicitly highlight the urgent need for novel theoretical frameworks. These frameworks should be firmly grounded in contemporary neuroscience and cognitive science, moving beyond simplistic brain function mappings or purely voluntary decision models. Future lines of research could explore how task failure varies in relation to different exercise intensities and modalities, with special attention to interindividual differences in physiological and perceptual responses, as well as in the development of psychological training strategies that strengthen mental resilience. It would also be valuable to conduct studies that individualize interventions based on each subject’s unique characteristics, allowing for personalized training in endurance sports and optimizing performance according to each athlete’s specific attributes.

## Footnotes

1 We know that perceived effort and perceived exertion are different concepts, but they are used interchangeably for the sake of simplicity.

## Declarations

### Availability of Data and Material

The data supporting this study’s findings are available from the corresponding author upon reasonable request.
